# Cingulate GABA levels inversely correlate with the intensity of ongoing chronic knee osteoarthritis pain

**DOI:** 10.1177/1744806916650690

**Published:** 2016-05-01

**Authors:** Diane Reckziegel, Felix Raschke, William J Cottam, Dorothee P Auer

**Affiliations:** 1 Arthritis Research UK Pain Centre, University of Nottingham, UK; 2 Sir Peter Mansfield Imaging Centre, University of Nottingham, UK; 3 Division of Clinical Neuroscience, Radiological Sciences, University of Nottingham, UK

**Keywords:** Osteoarthritis pain, magnetic resonance spectroscopy, γ-aminobutyric acid, anterior cingulate cortex, neurotransmission, pain chronification

## Abstract

**Background:**

This study aims to investigate the role of the mid-anterior cingulate cortex γ-aminobutyric acid levels in chronic nociceptive pain. The molecular mechanisms of pain chronification are not well understood. In fibromyalgia, low mid-anterior cingulate cortex γ-aminobutyric acid was associated with high pain suggesting a role of prefrontal disinhibition. We hypothesize that mid-anterior cingulate cortex GABAergic disinhibition may underpin chronic pain independent of the pain etiology and comorbid negative affect. Proton magnetic resonance spectra were acquired at 3T from the mid-anterior cingulate cortex in 20 patients with chronic painful knee osteoarthritis, and 19 healthy pain-free individuals using a point resolved spectroscopy sequence optimized for detection of γ-aminobutyric acid. Participants underwent questionnaires for negative affect (depression and anxiety) and psychophysical pain phenotyping.

**Results:**

No differences in mid-anterior cingulate cortex γ-aminobutyric acid or other metabolite levels were detected between groups. Ratings of perceived intensity of ongoing osteoarthritis pain were inversely correlated with γ-aminobutyric acid (*r* = −0.758, *p* < 0.001), but no correlations were seen for negative affect or pain thresholds. The pain γ-aminobutyric acid interrelation remained strong when controlling for depression (*r* = −0.820, *p* < 0.001). Combined levels of glutamine and glutamate were unrelated to psychometric or to pain thresholds.

**Conclusion:**

Our study supports mid-anterior cingulate cortex γ-aminobutyric acid as a potential marker of pain severity in chronic nociceptive pain states independent of negative affect. The findings suggest that GABAergic disinhibition of the salience network may underlie sensitization to averse stimuli as a mechanism contributing to pain chronification.

## Background

Osteoarthritis (OA) is a major cause of long-term disability and chronic pain in the elderly. Despite the primary nociceptive nature of OA pain resulting from joint tissue damage, the level of pain severity does not correlate well with the radiographic joint disease severity, suggesting the contribution of central nervous system mechanisms to pain progression in OA.^1,2^ The neural mechanisms underlying chronic OA pain are, however, poorly understood.

There is convergent evidence supporting a role of the mid-anterior cingulate cortex (mACC) in pain perception, intensity encoding, and pain progression.^3,4^ The ACC and especially mACC is one of the most commonly activated brain areas in experimental pain studies^5,6^ and also consistently reported as a pain intensity encoding region in healthy volunteers.^3,7–9^ Moreover, activity in the ACC appears to track pain intensity in ongoing chronic OA pain. Several groups found elevated baseline ACC activity in painful OA compared to healthy populations indexed as regional cerebral blood flow or glucose metabolism.^10,11^ We also showed that local blood flow in the dorsal ACC covaried with the reported intensity of spontaneous pain.^12^

Evidence from animal investigations indicate that the ACC can exert strong modulation of both affective and sensory aspects of pain via activation of the periaqueductal gray and rostral ventral medulla, as shown by electrophysiological recordings in combination with ACC stimulation and electrical stimulation or chemical activation and blockade.^5,13,14^ However, little is known about the molecular basis of altered neural processing in the ACC.^15–17^

GABA receptors are widely distributed in the spinal cord, thalamus, and cortex, both pre- and postsynaptically and are considered to modulate pain.^18–20^ The role of GABA in pain transmission could be demonstrated with baclofen, a GABA-B receptor agonist, being an effective analgesic in both preclinical models of acute and chronic pain.^19,21^ Advanced proton spectroscopic techniques allow non-invasive assessment of GABA levels, which indicate inhibitory neural activity.^22^ However, *in-vivo*, GABA detection is particularly challenging due to its low concentration in the brain and the substantial spectral overlap with more highly concentrated metabolites; therefore, sophisticated acquisition approaches such as spectral editing, J-coupling,^23^ or optimized sequences are needed.^24,25^

To date, few human studies obtained reliable GABA measurements in chronic pain by implementing such techniques at 3T.^26–29^ In painful trigeminal neuralgia, reduced GABA/Cr was detected in the thalamus,^28^ while in fibromyalgia and diabetic neuropathy insular GABA levels were decreased.^27,29^ In fibromyalgia, GABA correlated with pain sensitivity in the posterior insula^27^ and negatively with the magnitude of perceived pain in the ACC in a small group of patients (*n* = 10).^26^ Thermal pain was shown to induce GABA decreases in the occipital cortex but no significant changes were noted in the mACC in healthy volunteers.^21^ Conversely, a GABA increase was noted in the rostral ACC in healthy controls.^30^ Taken together, GABA may play a region and condition dependent modulatory role in pain perception and may underpin pain chronification.

It is conceivable that ACC GABA reduction may be a common mechanism of pain progression regardless of pain etiology. Interestingly, GABA concentrations were found to predict blood oxygenation level dependent (BOLD) responsiveness in several brain regions and networks^31,32^ and may thus explain enhanced ACC activity in chronic pain conditions. We hypothesize that GABAergic disinhibition (low GABA) in the nociceptive ACC is a molecular marker of ongoing pain severity in chronic painful OA.

Here, we studied the regional GABA levels in the mACC of individuals with chronic nociceptive knee OA pain and pain-free control subjects. We employed an optimized point resolved spectroscopy sequence (PRESS) for GABA detection. The sequence does not require use of special editing techniques, is less sensitive to macromolecule contamination than Mescher-Garwood (MEGA) PRESS and shows very good reproducibility at 3T.^24,33^ We hypothesize that mACC GABA is reduced in chronic OA pain and related to the severity of ongoing pain.

## Methods

The data presented here have been collected for this magnetic resonance spectroscopy (MRS) substudy as part of a larger multimodal investigation conducted in accordance with The Declaration of Helsinki, approved by the Nottingham Research Ethics Committee II (10/H0408/115) and the local Research and Innovation department.

### Participants

Participants with chronic knee OA pain and elderly, healthy, pain-free control participants were included. Controls were matched for age in the main study but not in the current MRS substudy. In order to address this, we additionally report age-matched subgroup results. In order to be included, patients were required to have radiographically diagnosed knee OA and pain during most of the day and for most of the days in the last month. Pain duration ranged from 1 to 24 years (mean ± SD, 7.7 ± 4.9 years). OA pain is largely considered nociceptive, but in order to exclude putative neuropathic pain phenotypes, patients with likely neuropathic-like syndromes were excluded using the PainDETECT screening tool (score 19 or higher).^34^ All participants provided written informed consent and underwent MRI, pain and affect questionnaires, and sensory pain phenotyping. The sample size was 39 (20 patients, age 67 ± 9 years, 11 males and 19 controls, age 59 ± 9 years, 7 males). Data from the original larger study will be published elsewhere.^12^

### Phenotypic and MRS data acquisition

Immediately before the scan session, participants rated the intensity of their spontaneous pain, as measured by using a 0 to 100 Visual Analogue Scale (VAS), to which participants were instructed to consider 0 as “no pain” and 100 as “excruciating pain.” Questionnaire evaluation included PainDETECT,^34^ the Pain Catastrophizing Scale (PCS),^35^ Beck Depression Inventory-II (BDI),^36^ and the State-trait Anxiety Inventory (STAI).^37^ All participants were psychophysically examined by means of pain pressure thresholds (PPT) in response to pressure algometer (Somedic AB, Sweden) stimulations of the medial aspect of the patella and at a site 3 cm inferior to the sternal notch. These were taken as examples of PPT local and remote to the affected joint.

MR data were acquired on a clinical 3T GE (General Electric, Milwaukee, WI) MR Discovery 750 Whole Body scanner equipped with a 32 channel head coil. Axial T1-weighted images were obtained using fast spoiled gradient echo, with echo time (TE) = 3.18 ms, repetition time (TR) = 8.2 ms, in-plane matrix: 256 × 256, voxel size 1 × 1 × 1 mm^3^, scan time: 4 minute. Single voxel spectra from the ACC were collected using a PRESS sequence optimized for GABA detection^24^ with TE = 105 ms, TE_1_ = 15 ms, TR = 2.5 s, 128 averages, 16 water unsuppressed averages for water scaling, scan time: 6 minute.

**Figure 1.**
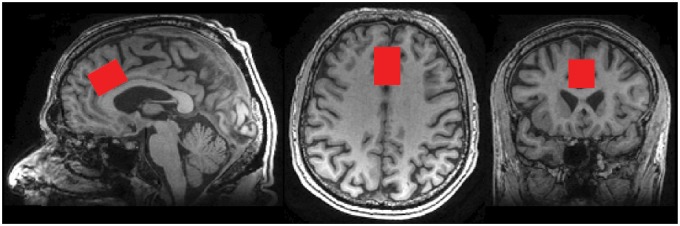
Typical MRS voxel position (red box) superimposed on anatomical T1-weighted image.

The volume of interest (VOI) was positioned in the mid-ACC, on the axial and coronal views, parallel to the body of the corpus callosum on the sagittal view (Figure 1), with a typical MRS volume of 12 ml, although in eight cases voxel volume varied between 8 and 10 ml.

### Data analysis

All MRS data were processed offline using MATLAB (MathWorks Inc., Natick, MA, USA). For each subject, the 128 individual spectra were averaged by the scanner in blocks of 16 spectra, equivalent to eight phase cycles. To correct for motion-induced frequency drifts, the resulting eight spectra for each subject were realigned to the N-acetylaspartate (NAA) peak by fitting a Lorentzian line shape.

**Figure 2.**
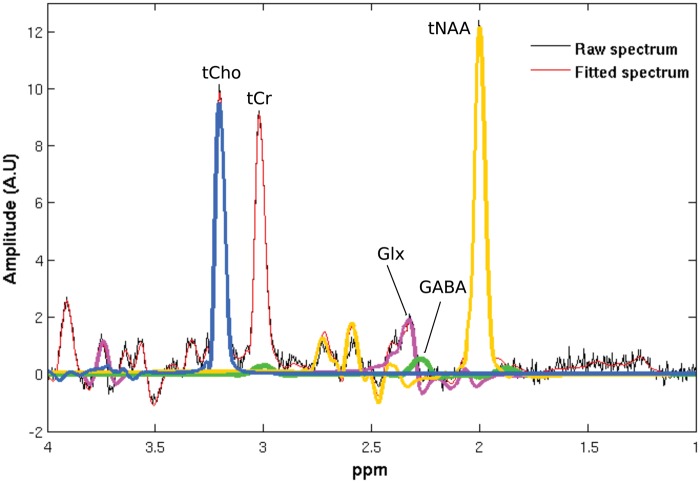
Example MR spectrum and LCModel derived metabolite fit of components using GABA optimized PRESS. Green: GABA; purple: Glx, total glutamate and glutamine; blue: tCho, total choline-containing moieties; yellow: tNAA, total N-acetyl-asparatate; tCr: total creatine; ppm: parts per million; A.U.: arbitrary units.

Metabolite levels were estimated using the LCModel v6.3^38,39^ and a simulated basis set (Figure 2).^24^ The unsuppressed water signal was used for water scaling.

The minimal standard deviation of the fitting was estimated using the Cramér-Rao Lower Bound (CRLB), expressed as the percentage of estimated metabolite levels. Although CRLB < 20% is a commonly used criterion for quality control of MR spectroscopy data, recent studies argue that such filtering may lead to erroneous or missed statistical findings, especially when studying conditions with reduction of GABA.^40,41^ Therefore, this criterion was not utilized and instead the full width at half maximum (FWHM) of the water peak, which is particularly relevant for decomposition of overlapping peaks was used for quality control. Data sets with FWHM > 0.078 ppm (>10 Hz) were excluded from the analysis.

All metabolite values quantified by LCModel were corrected for differences in tissue water concentrations in white matter (WM), gray matter (GM), and cerebral spinal fluid (CSF).^42^ For this purpose, the T1-weighted images were segmented into WM, GM, and CSF using SPM8 (Statistical Parametric Mapping version 8, London, UK). An in-house MATLAB script was used to create a mask for the MRS VOI and calculate the volume fractions of WM (f_wm_), GM (f_gm_), and CSF (f_csf_) within the VOI. The corrected water concentration (WCONC_corr_) was estimated within the VOI,^38^ according to:

$${\text{WCONC}}_{\text{corr}}=43300{\text{f}}_{\text{gm}}+35880{\text{f}}_{\text{wm}}+{55556\text{f}}_{\text{csf}}$$

Since metabolite concentrations within CSF can be assumed to be negligible,^43^ a partial volume correction factor can be added to the equation above, in order to correct for this:

$${\text{WCONC}}_{\text{corr}}=(43300{\text{f}}_{\text{gm}}+35880{\text{f}}_{\text{wm}}+{55556\text{f}}_{\text{csf}})/(1-{\text{f}}_{\text{csf}})$$

LCModel by default sets the water concentration reference to 35,880 mM (equivalent to pure WM), hence default LCModel metabolite outputs M_LCM_ were corrected for the newly calculated WCONC_corr_ as

$${\text{M}}_{\text{corr}}={\text{M}}_{\text{LCM}}{\text{WCONC}}_{\text{corr}}/35880$$

No correction for relaxation times was performed on the data, and the metabolite levels are therefore reported in arbitrary units.

### Statistical analysis

All statistical analyses were performed using IBM SPSS Statistics 22. Metabolites were described using mean and standard deviation. Normal distribution of metabolite data was determined by visual inspection of histogram plots.

Differences in neurochemical levels between patient and control groups were tested with independent samples *t* tests (*p* < 0.05 set a priori as significant). Even though it has been previously shown that there are no gender or age differences in GABA in the ACC,^44^ a second group comparison was undertaken after exclusion of three patients and three controls in order to improve age matching (*n* = 14 vs. 14). Pearson’s product moment correlation coefficient, *r*, was used for continuous, approximately normally distributed variables. Other correlations were tested using the Spearman’s rank correlation method, ρ, with values of *p* < 0.05 considered statistically significant. Partial correlation was also used to control for potential confounding factors.

## Results

Two data sets were excluded due to FWHM > 10 Hz, and three were not included because movement caused significant deviation of the MRS VOI from the planned location as determined relative to the structural T1-weighted scan. This resulted in 17 chronic knee OA pain patients (11 males, age range: 48 to 83 years) and 17 healthy controls (7 males, age range: 43 to 72 years).

All data sets had CRLBs below 25% for GABA, NAA, NAA plus N-acetylaspartyl-glutamate (NAA + NAAG), glutamate (Glu), Glx (Glu + glutamine), myo-inositol (Ins), and total choline (tCho). There were no significant differences in tissue proportions in the VOI between patients and controls (main group or age-matched subgroups). GABA did not show correlations with tissue proportions.

Depression scores, as measured by the BDI, were higher in the patient population (*t* = 3.1, *p* < 0.005), while state and trait anxiety, pain catastrophizing, and PPT did not differ. Spontaneous pain intensity ratings prior to the scan session, as measured on the VAS, ranged from 0 to 80 in the patient group (mean ± SD: 31 ± 28) Medication intake details are specified in the Supplementary Table (see supplementary material).

No significant differences in mACC GABA (mean ± SD, 4.13 ± 0.75 arbitrary units (a.u.) for patients, 4.25 ± 1.16 a.u. for controls) or Glx (mean ± SD, 16.46 ± 2.8 a.u. for patients, 16.99 ± 1.96 a.u. for controls) were noted. To confirm these results, three healthy and three OA participants were excluded for improved age-matching, resulting in 14 patients (age range 48 to 77 years, mean: 64 years) and 14 controls (age 51 to 72 years, mean: 62 years) (Table 1). Still, when comparing the age-matched groups, no differences in mean levels of GABA or Glx were detected. There were also no group differences in any other measured metabolite levels (Table 2).

**Table 1.** Age-matched group comparisons and demographics.

	OA (mean ± SD)	HV (mean ± SD)	*t* test
Subjects	14	14	–
Age (years)	64.1 ± 7.4	62.0 ± 6.6	NS
Gender	9 males	5 males	–
Pain duration (years)	7.7 ± 5.1	0	–
BDI	6.2 ± 5.0	1.3 ± 1.6	3.4, *p* < 0.005
STAI-S	31.1 ± 9.7	25.0 ± 6.4	NS
STAI-T	33.2 ± 8.5	29.2 ± 6.0	NS
PCS	16.5 ± 11.4	11.4 ± 9.1	NS
PPT remote (kPa)	260 ± 146	217 ± 90	NS
PPT local (kPa)	242 ± 118	334 ± 147	NS
VAS	29.0 ± 28.4	–	–

OA: osteoarthritis patients; HV: healthy volunteers; VAS: Visual Analogue Scale; PCS: Pain Catastrophizing Scale; BDI: Beck Depression Inventory II; PPT: Pressure Pain Thresholds; STAI: State Trait Anxiety Inventory (-S, state and -T, trait); NS: Not significant.

**Table 2.** Summary of metabolite levels for age-matched subgroups, given in arbitrary units.

Metabolite	OA (mean ± SD)	HV (mean ± SD)	*t* test
GABA	4.26 ± 0.76	4.28 ± 1.21	NS
Glx	17.11 ± 2.70	17.08 ± 2.11	NS
NAA	25.87 ± 2.67	26.78 ± 3.04	NS
tCho	5.99 ± 1.23	5.91 ± 0.73	NS
Ins	11.67 ± 2.02	12.01 ± 1.40	NS

OA: osteoarthritis patients; HV: healthy volunteers; GABA: γ-aminobutyric acid; Glx: total glutamate and glutamine; NAA: N-acetylaspartate; tCho: total choline; Ins: myo-inositol.

**Figure 3.**
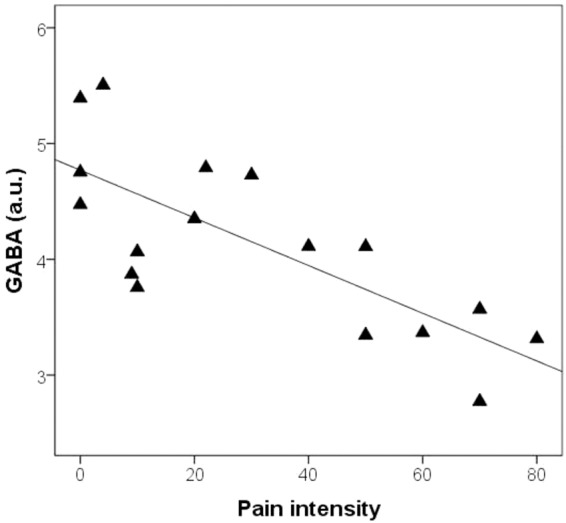
Scatterplot of mACC GABA levels and ongoing osteoarthritis pain intensity.

Pain intensity ratings in the OA group correlated negatively and strongly with GABA (*r* = −0.758, *p* < 0.001) (Figure 3), but not Glx (Table 3). Depression, catastrophizing, and anxiety scores as well as PPT did not relate to GABA (Table 3). Since VAS was only approximately normally distributed, we verified the association with GABA using Spearman correlation (ρ = −0.708, *p* < 0.005).

**Table 3.** Correlations for OA group (*n* = 17), HV group (17) and all (*n* = 34).

Variable 1	Variable 2	Controlling for	Correlation (OA)	Correlation (HV)	Correlation (all)
GABA	VAS	–	*r* = −0.758, *p* < 0.001		
GABA	VAS	Volume	*r* = −0.739, *p* < 0.005	–	–
GABA	VAS	BDI	*r* = −0.820, *p* < 0.001	–	–
GABA	VAS	Age	*r* = −0.712, *p* < 0.005	–	–
GABA	VAS	Gender	*r* = −0.757, *p* < 0.005	–	–
GABA	VAS	Age, gender	*r* = −0.711, *p* < 0.005		
Glx	VAS	Volume	NS	–	–
Glx	VAS	Age	NS	–	–
Glx	VAS	Gender	NS	–	–
GABA	PCS	–	NS	NS	NS
GABA	PPT	–	NS	NS	NS
GABA	BDI	–	NS	NS	NS
GABA	STAI	–	NS	NS	NS

OA: osteoarthritis patients; HV: healthy volunteers; GABA: γ-aminobutyric acid; Glx: Glu + glutamine; VAS, Visual Analogue Scale; PCS, Pain Catastrophizing Scale; BDI, Beck Depression Inventory; PPT, Pressure Pain Thresholds; STAI, State Trait Anxiety Inventory; NS: non-significant.

GABA did not correlate with age and when controlling for both age and gender, the GABA and VAS correlation was kept (*r* = −0.711, *p* < 0.005). The correlation was also kept when controlling for depression scores (*r* = −0.820, *p* < 0.001) (Table 3).

## Discussion

Using an optimized spectroscopic sequence for GABA detection, we found that GABA levels in the mid-ACC in patients with chronic knee OA pain were closely negatively correlated with perceived clinical pain severity. This association was independent of negative affect.

We found that patients reporting more severe ongoing knee OA pain showed the lowest mid-cingulate GABA levels, suggesting that there is more ACC disinhibition in those with higher levels of pain. Since the mid-ACC is a hub of the salience network,^45^ low GABA tone*,* *i.e.* disinhibition, would result in increased salience network activity. This in turn could explain enhanced pain perception as noted in chronic OA^46^ and may offer a molecular mechanism of pain progression. A very similar association between mACC GABA and reported pain severity has been recently reported in fibromyalgia.^26^ Fibromyalgia is, however, considered a central pain disorder with common comorbidities and yet to be determined pathomechanism, with controversial classification as neuropathic pain.^47,48^ By contrast, OA is considered a primary nociceptive pain disorder despite good evidence for development of central pain components and neuropathic features in a subgroup of about 5% of patients.^49^ We, therefore, excluded patients with neuropathic features based on PainDETECT^34^ and further explored whether the observed interrelation between GABA and pain severity was modulated by negative affect. Controlling for negative mood scores that were elevated in our OA patient cohort did not affect the correlation resulting in notionally higher (67%) mutually explained variance. The independent strong association suggests that the link of low GABA with more severe OA pain is not a reflection of comorbid negative affect but more likely a feature of central pain augmentation. The close similarity with findings in fibromyalgia furthermore point to shared mechanisms between a predominately nociceptive and a primarily central pain disorder.

Our findings also support a body of fMRI and PET literature in healthy volunteers which suggest certain parts of the ACC as a pain intensity encoding area during painful stimulation.^3,7–9,50^ Also, in OA, it has been previously shown that local blood flow in the dorsal ACC covaried with the reported intensity of spontaneous pain.^12^ Taken together, mACC GABA levels might index the pain state in inverse relation.

Notably, this inverse covariation of GABA with pain intensity points to underlying alterations in the presynaptic terminals, synaptic vesicles, or in GABA uptake mechanisms.^22^ The methodology utilized here is not capable of discerning these possibilities; however, our results are consistent with functional neuroimaging findings in OA. A number of previous studies have shown inverse correlations of GABA concentrations with BOLD activity both during task and rest, locally and at the network level.^31^ In particular, one study found that GABA and Glu in the posteromedial cortex, a node of the default mode network, predict intrinsic functional connectivity of the network, as determined by positive GABA correlation and negative Glu correlation.^51^ Default mode and salience network activity is largely anticorrelated; hence low GABA may be linked with upregulated salience network activity. In fact, there is substantial evidence from fMRI studies suggesting aberrant salience network activity in chronic pain conditions.^52^ Further studies are warranted to investigate whether mACC GABA predicts salience network activity and if such network upregulation may mediate the association with chronic pain.

Interestingly, we did not find any Glx abnormalities or interrelation with pain characteristics. As both glutamatergic and GABAergic transmission may modulate neuronal activity, the observed increased ACC activity in some functional neuroimaging studies in ongoing chronic pain may be related to inhibitory or excitatory neurotransmission. The lack of Glx interrelation with reported pain, however, suggests that pain augmentation in nociceptive knee OA pain is predominantly linked to GABAergic disinhibition. This would be in partial disagreement with the established role of glutamatergic neurotransmission in the development of neuropathic pain in preclinical models.^53^ Blockage of excitatory neurotransmission in the ACC has been shown to inhibit pain-like behavior in animals.^54^ Also, a recent study in healthy volunteers detected an increase in ACC Glu concentrations in response to thermal stimuli.^21^

In fibromyalgia patients, a study using transcranial direct current stimulation (tDCS) detected ACC Glx modulation following active tDCS and that baseline levels of ACC Glx levels were associated with pain response.^26^ ACC Glx concentrations have not been reported in fibromyalgia in comparison with healthy state.^27^ In addition to Glx, some chronic pain studies also found a concomitant inverse relation in insular GABA concentrations, such as Petrou et al.^29^ in diabetic neuropathy, or only reduced insular GABA, as detected in fibromyalgia,^27^ raising the possibility of an increase in the insular Glx/GABA ratio as an underlying mechanism in some clinical pain states.^55^ When testing for ACC Glx/GABA differences between our two cohorts, we did not detect abnormalities, indicating that, at least in the mACC, the ratio imbalance is not present in chronic OA pain. Taken together, our findings suggest a less prominent role of mACC excitation in nociceptive pain augmentation.

The suggested predominant role of GABA induced disinhibition and lack of evidence for enhanced excitatory activity (no Glx change) highlight a promising role of mACC GABA as a mechanistic biomarker of chronic OA pain. The increasing availability of 3T MRI scanners with dedicated GABA detection sequences underline the potential of mACC GABA as novel molecular pain biomarker with particular relevance for evaluation of GABAergic modulation in pain relief. Patients with high levels of pain and therefore low concentrations of mACC GABA could potentially be more responsive to interventions that would enhance GABAergic neurotransmission such as gabapentin and transcranial magnetic stimulation.^22,56^ Moreover, the feasibility to non-invasively measure mACC GABA provides unique opportunities to re-evaluate the mechanistic efficacy of pharmacological GABA modulation in the search of novel, more potent analgesics.

Limitations to the present experiment include the relatively small sample size; however, it is larger than previously published GABA investigations of pain. Potential differences between groups may have passed unnoticed due to the relatively large voxel size and lack of gender matching. Our main aim was to evaluate the role of GABA in chronic knee OA pain. Given the low concentration of GABA in the brain, its detection by proton MR spectroscopy is challenging and, consequently, the literature in pain research is scarce. Therefore, we employed an acquisition technique tailored at GABA, which consists of a PRESS sequence that has been optimized for reliable GABA detection.^24^ Relative to the most commonly used MEGA-PRESS technique,^23^ the method utilized here yields GABA measurements with less contribution from macromolecules, requires less acquisition time and is less susceptible to subject motion.^24,33^ A drawback, however, is that most published GABA literature tends to use MEGA-PRESS for higher specificity to GABA, making direct quantitative comparisons less straightforward. Another limitation of the present study is the lack of use of an analgesic to prove whether mid ACC GABA indeed increases with pain reduction. Future studies performed at higher field strengths such as 7T may be particularly advantageous, not only in order to decrease voxel sizes, given the increased signal to noise ratio and, therefore, allow disclosure of more region-specific neurochemical information but also to measure multiple voxels.

## Conclusions

In conclusion, this study provides further evidence of the role of the mid-ACC for ongoing clinical pain and suggests reduced GABAergic tone as a putative underlying molecular mechanism underpinning chronic pain. The strong inverse relation of GABA with spontaneous OA pain ratings were independent of negative affect and supports use of GABA as a biomarker for mechanistic evaluation of GABAergic modulation for pain relief in chronic OA pain.

## Supplementary Material

Supplementary material
