# Supplementary material for: Cingulate GABA levels inversely correlate with the intensity of ongoing chronic knee osteoarthritis pain
Source: Mol Pain. 2016 May 1;12:1744806916650690. doi: 10.1177/1744806916650690 (PMC4956171; doi:10.1177/1744806916650690)
Supplement: Supplementary material [file MPX650690_supplementary_material.pdf]

Supplementary Material

for

Cingulate GABA levels inversely correlate with the intensity of ongoing chronic knee osteoarthritis pain

Diane Reckziegel, Felix Raschke, William J Cottam, Dorothee P Auer

Supplementary Table

ID	Group	Medication
1	HV	none
2	HV	none
3	HV	premarin
4	HV	none
5	HV	none
6	HV	none
7	HV	none
8	HV	none
9	HV	none
10	HV	none
11	HV	none
12	HV	none
13	HV	none
14	HV	paracetamol, losartan, bendroflumethiazide, omeprazole
15	HV	fexfenadin
16	HV	none
17	HV	remipril, bendroflumethiazide, amlodipine
18	OA	NA
19	OA	none
20	OA	none
21	OA	none
22	OA	glucosamine, bendroflumethiazide, simvastatin, contiflow
23	OA	amlodipine
24	OA	none
25	OA	none
26	OA	none
27	OA	none
28	OA	none
29	OA	none
30	OA	paracetamol, losartan
31	OA	NA
32	OA	NA
33	OA	NA
34	OA	none

List of medications taken less than twenty-four hours before the scanning session.
